# Retinoids for the Treatment of Refractory Grover’s Disease: A Case Series and Review of the Literature

**DOI:** 10.7759/cureus.53510

**Published:** 2024-02-03

**Authors:** David Moodie, Charles Dunn, Chloe Fernandez, Rajiv Nathoo

**Affiliations:** 1 Medicine, University of Central Florida College of Medicine, Orlando, USA; 2 Dermatology, Kansas City University-Graduate Medical Education Consortium/Advanced Dermatology and Cosmetic Surgery, Oviedo, USA

**Keywords:** isotretinoin, acitretin, retinoids, grover’s disease, transient acantholyitic dermatitis

## Abstract

Grover’s disease, also known as transient acantholytic dermatosis (TAD), currently has no published randomized control trials regarding the treatment of the disease; thus, evidence for treatment is largely derived from case studies and case reports. In this case series, we summarize the current treatment options for Grover’s disease and discuss two cases of refractory Grover’s disease treated with low-dose oral isotretinoin in patients who previously failed to reach clearance with multiple treatment options. Our aim is to highlight the efficacy of low-dose systemic retinoid therapy in Grover's disease when other treatment options prove unsatisfactory.

## Introduction

Grover’s disease, also known as transient acantholytic dermatosis (TAD), is a benign papulovesicular pruritic eruption primarily distributed on the trunk and extremities. The disease typically presents with a waxing and waning course; however, it is not uncommon for severe, generalized cases to prove refractory to standard therapeutics. Currently, there are no published randomized controlled trials for the treatment of Grover’s disease, and evidence for treatment is largely derived from case studies and case reports documenting successful treatment. First-line therapy generally includes high-potency topical corticosteroids, moisturizers, and emollients with antihistamines to help reduce pruritus. In more severe cases proving resistant to first-line therapy, treatments using low-dose systemic corticosteroids, low-dose systemic retinoids, and photochemotherapy have proven effective [1,2]. In this manuscript, we report two cases of patients with severe, refractory TAD who responded dramatically to systemic retinoid treatment. Our aim with this manuscript is to summarize the existing treatment options for TAD and highlight the efficacy of low-dose retinoid therapy when other treatments prove unsatisfactory.

## Case presentation

Case one

Patient one, a 40-year-old female with a medical history of depression, anxiety, and guttate psoriasis, presented to the clinic with biopsy-proven TAD. The patient failed to reach clearance with topical corticosteroids, oral corticosteroids, and oral antihistamines over 18 months. She endorsed significant pruritus and the psychosocial impact of the disease process on her personal and professional lives. She was started on 40 mg of oral isotretinoin daily and achieved complete clearance of her Grover’s disease after a single month of therapy. She completed a cumulative isotretinoin course of 123 mg/kg with no reported adverse effects. She is currently six months post-completion of retinoid therapy with sustained disease remission (Figure I).

**Figure 1:**
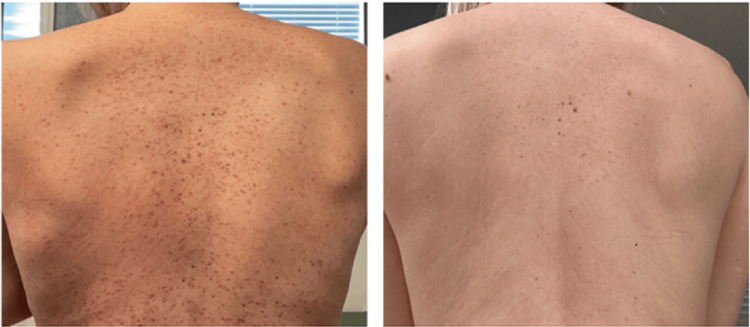
Patient one before (left) and after (right) a five-month treatment course with isotretinoin

Case two

**Figure 2:**
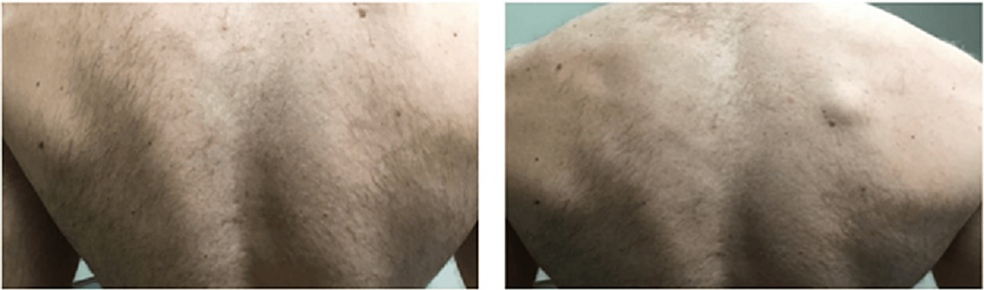
Patient two, before (left) and after (right), 11-month treatment course with isotretinoin

Patient two, a 45-year-old male with a medical history of asthma, was diagnosed with TAD by biopsy in April 2022. The patient failed to reach clearance with topical corticosteroids and over-the-counter moisturizers. He was started on 40mg of oral isotretinoin daily for five months, 40 mg twice daily for an additional four months, and back down to 40mg daily for the remaining two months of his 11-month treatment course (cumulative treatment dose: 220 mg/kg). The patient was seen at the end of the fourth month for a follow-up with complete clearance of his rash noted. Clobetasol was prescribed for breakthrough lesions, which did not occur after six months of therapy. At his two-month appointment following the conclusion of therapy, he demonstrated no residual lesions and significant satisfaction with his therapeutic course (Figure 2).

## Discussion

These cases highlight the presentation of Grover’s disease in patients with a recalcitrant disease course. Grover’s disease, or TAD, classically presents as a self-limiting, papulovesicular pruritic rash with a primarily truncal distribution [3]. The face, palms, and soles are typically spared. Though clinically distinct, Grover's disease mimics Darier disease, Hailey-Hailey disease, and pemphigus vulgaris histopathologically [4].

While the etiology of this disease is poorly understood, several triggers have been proposed, including heat, sweating, medications, infections, and malignancies. Non-pharmacological management of TAD involves avoiding suspected triggers when possible [5]. High-potency topical corticosteroids, moisturizers, and emollients are used as first-line therapies [5]. Oral antihistamines may be added to help reduce pruritus. Low-dose systemic corticosteroids, low-dose systemic retinoids, or photochemotherapy have been effective as treatments for patients with persistent, recalcitrant Grover’s disease [1, 2]. Because there are no published randomized controlled trials on the treatment of TAD at this time, evidence for treatment, shown in Table 1, has come through case reports and case studies.

**Table 1:** Therapies for transient acantholytic dermatosis (Grover’s disease) Source: [5]

Topical therapies	Systemic therapies	Alternate therapies
Emollients/Moisturizers	Retinoids (acitretin, isotretinoin, etretinate)	Phototherapy
Corticosteroids	Corticosteroids	Photochemotherapy
All trans-retinoic acid	Methotrexate	Photodynamic therapy
Vitamin D analogues (calcipotriol)	Etanercept	Chemical Peel
	Vitamin A	
	Antibiotics (erythromycin, clindamycin)	

The use of retinoids, such as isotretinoin and acitretin, has emerged as a novel approach to the treatment of persistent, recalcitrant TAD. Retinoids are synthetic vitamin A analogues that normalize cell proliferation and epidermal turnover in addition to exhibiting immune-modulating effects [6]. Through these mechanisms, and possibly others, retinoids prevent the underlying pathophysiology and acantholytic process that lead to the symptoms of Grover’s disease. Evidence of their contributions to the long-term remission and resolution of Grover's disease is demonstrated by the cases listed in Table 2.

**Table 2:** Use of retinoids in the treatment of transient acantholytic dermatosis

Study of case report/case series	Treatment	Recurrence post-treatment with systemic retinoid
Grover's disease treated with isotretinoin: Report of four cases [2]	Case 1: Isotretinoin 40 mg	Case 1: No recurrence 10 months post therapy
	Case 2: Isotretinoin 40 mg	Case 2: No recurrence 5 months post therapy
	Case 3: Isotretinoin 40 mg	Case 3: Relapse occurred 3 weeks post therapy
	Case 4: Isotretinoin 40 mg	Case 4: No relapse 5 months post therapy
Persistent generalized Grover disease: complete remission after treatment with oral acitretin [7]	Oral acitretin 0.8 mg/kg/day	No recurrence 26 months post therapy
Grover's disease: successful treatment with acitretin and calcipotriol [8]	Topical calcipotriol and low dose acitretin	No recurrence post therapy noted

As one of the largest case series to date on the treatment of TAD with low-dose systemic retinoids, we hope that these cases raise awareness about this relatively rare disease and effective treatment options for persistent, recalcitrant courses of the disease. With a limited body of research on treatment options, we believe focusing on systemic retinoids for refractory cases of the disease is of great importance for future researchers and the advancement of treatment options. Our findings in these cases, along with other case reports and case series, will hopefully lay the groundwork for more comprehensive clinical trials in the future to help establish systemic retinoids as a first-line therapeutic option for patients with refractory TAD.

## Conclusions

These reports illustrate an example of low-dose oral isotretinoin as a safe option for patients with recalcitrant transient acantholytic dermatosis who have failed traditional therapies. Because there are currently no randomized controlled trials for the treatment of TAD, treatment has been based on case reports and case series. This case series is one of the largest-to-date demonstrations of the successful use of systemic retinoids in the treatment of severe, refractory cases of TAD. Despite the etiology and pathophysiology of TAD being poorly understood, there are a growing number of cases demonstrating systemic retinoids to be an effective treatment for persistent cases of TAD, and further studies should be conducted to examine the relationship between vitamin A analogues and the underlying pathophysiology of Grover’s disease.
